# Effectiveness and tolerability of eptinezumab in treating patients with migraine resistant to conventional preventive medications and CGRP (receptor) antibodies: a multicentre retrospective real-world analysis from Germany

**DOI:** 10.1186/s10194-024-01788-1

**Published:** 2024-05-16

**Authors:** Armin Scheffler, Pauline Wenzel, Merle Bendig, Astrid Gendolla, Jale Basten, Christoph Kleinschnitz, Michael Nsaka, Diana Lindner, Steffen Naegel, Philipp Burow, Robert Fleischmann, Dagny Holle

**Affiliations:** 1 Department of Neurology and Centre for Translational Neuro- and Behavioral Sciences (C-TNBS), West German Headache Centre, University Hospital Essen, University Duisburg-Essen, Hufelandstr. 55, Essen, 45147 Germany; 2 Department of Neurology, University of Greifswald, Greifswald, Germany; 3 Praxis Gendolla, Essen, Germany; 4 Department of Medical Informatics, Biometry and Epidemiology, Ruhr University Bochum, Bochum, Germany; 5 Department of Neurology, Martin-Luther-University Halle-Wittenberg, University Hospital Halle, Halle (Saale), Germany; 6 Department of Neurology, Alfried-Krupp Krankenhaus, Essen, Germany

**Keywords:** Episodic migraine, Chronic migraine, Drug resistant, Antibody switch

## Abstract

**Background:**

Eptinezumab is a monoclonal antibody that targets calcitonin gene-related peptide (CGRP mAb) and is used for migraine prophylaxis. Efficacy data are mainly from clinical trials, real-world data are hardly available yet. Reimbursement policy in Germany leads to eptinezumab mainly being used in patients having failed pre-treatment with other CGRP mAb. To date, it is unclear whether eptinezumab is efficacious and well tolerated in this population and how the treatment response differs from patients who are naive to CGRP mAbs.

**Methods:**

We analysed clinical routine data of 79 patients (episodic migraine (EM): *n* = 19; chronic migraine (CM): *n* = 60) from four different centres in Germany. All patients were treated with eptinezumab (100mg). Differences in monthly headache (MHD), migraine (MMD) and acute medication days (AMD) after three months were analysed. The correlation of response with the number of CGRP mAb failures was evaluated. Significance level has been corrected (alpha = 0.017).

**Results:**

After three months MHD, MMD and AMD were significantly reduced. In EM, the median reduction for MHD was 4.0 days (IQR: -6.5 to -1.0; *p* = 0.001), for MMD 3.0 days (IQR: -5.5 to -1.5; *p* < 0.001) and for AMD 2.0 days (IQR: -5.0 to -0.5; *p* = 0.006). In CM, median reduction of MHD was 4 days (IQR: -8.0 to 0.0; *p* < 0.001), 3.0 days (IQR: -6.0 to-1.0; *p* < 0.001) for MMD and 1.0 day (IQR: -5.0 to 0.0; *p* < 0.001) for AMD. All patients were resistant to conventional preventive therapies and most to CGRP mAbs. Fourteen patients had never received a CGRP mAb and 65 patients had received at least one mAb without sufficient effectiveness and/or intolerability (one: *n* = 20, two: *n* = 28, three: *n* = 17). There was a significant association between the number of prior therapies and the 30% MHD responder rate (none: 78.6%, one: 45.0%, two: 32.1%, three: 23.5%, *p* = 0.010). Regarding tolerability, 10.4% (8/77) reported mild side effects.

**Conclusions:**

The effectiveness of eptinezumab is significantly reduced in patients who have not previously responded to other CGRP mAbs. However, limitations such as the retrospective nature of the analysis, the small sample size and the short treatment period with only the lower dose of eptinezumab must be considered when interpreting the results.

## Introduction

Currently, there are four monoclonal antibodies (mAb) available for the preventive treatment of migraine: eptinezumab, fremanezumab, galcanezumab, and erenumab. These antibodies target calcitonin gene-related peptide (CGRP) or its receptor. Due to cost and reimbursement issues, these are usually used in patients who have not responded to non-specific migraine therapy. It is important to note that the use of these mAbs has been mainly limited to refractory patients.

Eptinezumab is the latest CGRP mAb authorized by the European Medicines Agency and the only mAb administered intravenously, which may offer benefits such as rapid onset of action and higher peak plasma levels. The efficacy of eptinezumab has been demonstrated in large controlled trials [1–3] including patients with up to four prior non-specific preventive therapies [4, 5] and medication overuse [6, 7]. However, its effectiveness in highly resistant patients who failed all eligible preventive treatments remains unclear. Furthermore, it has not yet been investigated whether eptinezumab is effective if other CGRP mAbs had been unsuccessful in the past.

Currently, there is very limited research on the effectiveness of drug resistant migraine patients [8] and on switching to a different CGRP mAb after discontinuing the first therapy due to lack of efficacy or poor tolerability [9]. The feasibility of switching to a third or fourth CGRP antibody remains unclear. This issue has especially not yet been explored in relation to eptinezumab.

This study examines the effectiveness and tolerability of eptinezumab in a real-world setting, with a particular focus on whether prior CGRP mAb therapy affects the response to therapy.

## Methods

A retrospective analysis was conducted on clinical routine data which included headache diaries, questionnaires, and medical records. Data on monthly headache days (MHD), monthly migraine days (MMD) and monthly days of acute drug intake (AMD) at baseline and three months after treatment was collected. The study included patients from four headache specialized centres in Germany and was conducted between October 2022 and December 2023 at the West German Headache Centre (Department of Neurology, University Hospital Essen), Practice Gendolla (Essen), University Hospital Halle (Saale, Department of Neurology) and University of Greifswald (Department of Neurology). The analysis was approved by the independent ethics committee of the University Hospital Essen (19–9004-BO). As this was a retrospective analysis of internal routine data, written informed consent was not required.

The patients were categorized as having either episodic migraine (EM) or chronic migraine (CM) based on the ICHD-3 criteria [23]. Furthermore, patients with a high frequency of MHD (between 15 and 30) and an MMD range of five to less than eight were also classified as CM patients due to the more difficult differentiation between migraine and headache days of chronic patients in the daily routine. Patients were defined as ptCGRP (previous treatment with CGRP mAb) when they received between one and three prior CGRP mAb treatments (erenumab, galcanezumab, fremanezumab) without sufficient effectiveness or having discontinued treatment due to intolerability. If patients did not receive any CGRP mAb in the past, they were defined as naïve.

All patients were administered 100mg of eptinezumab and had previously tried at least four approved non-specific prophylactic drugs (when EM) and five (when CM) without experiencing sufficient treatment effects, had discontinued those due to side effects or were ineligible for intake due to contraindication. The approved drug classes for treatment included betablockers (metoprolol or propranolol), tricyclic antidepressants (amitriptyline), calcium channel blockers (flunarizine), anticonvulsants (topiramate), and for CM, onabotulinumtoxin A. Thus, all patients were defined as drug resistant. Additionally, pre-treatment with CGRP mAb such as erenumab, galcanezumab, or fremanezumab was possible. It is important to note that small molecule CGRP antagonists were not available in Germany during this study, so pre-treatment with gepants was not applicable.

Wilcoxon-signed-rank test was used to test differences between baseline and three-month treatment outcomes for MHD, MMD, and AMD. To assess differences in treatment effectiveness between patients who had no ptCGRP and those with ptCGRP, responder rates were analysed using Pearson's Chi-squared test. Bonferroni correction was performed for multiple testing (adjusted significance level: α = 0.017). Patient satisfaction and side effects were evaluated descriptively when available. The analysis and visualisation were performed using R (version 4.3.2) and Office Professional Plus 2019 (Microsoft Corporation, Redmond, Washington, USA).

## Results

**Fig. 1:**
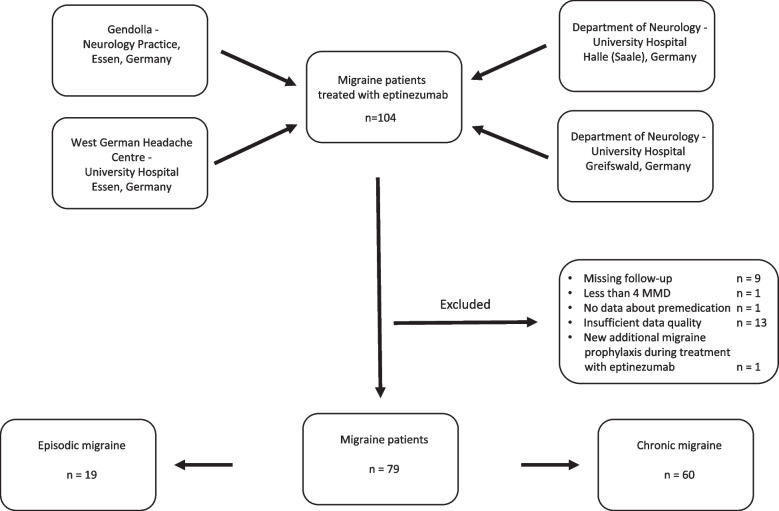
Patients included in study

Data of 104 patients treated with eptinezumab were collected, 79 patients (19 with EM and 60 with CM) could be included in the analysis. The reasons for exclusion are shown in Fig. 1.

Patients' characteristics and premedication including CGRP mAb are summarised in Table 1.

**Table 1:** Patients´ characteristics

	**Chronic (*N* = 60)**	**Episodic (*N* = 19)**	**Total (*N* = 79)**
**Age**			
Mean (SD)	46.4 (12.9)	50.9 (8.6)	47.5 (12.1)
Range	21.0—82.0	35.0—66.0	21.0—82.0
**Sex**			
Male	7 (11.7%)	2 (10.5%)	9 (11.4%)
Female	53 (88.3%)	17 (89.5%)	70 (88.6%)
**Aura**			
Yes	23 (38.3%)	7 (36.8%)	30 (38.0%)
No	37 (61.7%)	12 (63.2%)	49 (62.0%)
**MOH before treament**	34 (56.7%)	0 (0.0%)	34 (43.0%)
**CGRP antibodies before eptinezumab (detail)**			
None	10 (16.7%)	4 (21.1%)	14 (17.7%)
Erenumab	8 (13.3%)	2 (10.5%)	10 (12.7%)
Galcanezumab	3 (5.0%)	2 (10.5%)	5 (6.3%)
Fremanezumab	3 (5.0%)	2 (10.5%)	5 (6.3%)
Galcanezumab & Fremanezumab	3 (5.0%)	0 (0.0%)	3 (3.8%)
Galcanezumab & Erenumab	7 (11.7%)	1 (5.3%)	8 (10.1%)
Fremanezumab & Erenumab	11 (18.3%)	6 (31.6%)	17 (21.5%)
Fremanezumab & Erenumab & Galcanezumab	15 (25.0%)	2 (10.5%)	17 (21.5%)
**CGRP antibodies before eptinezumab (count)**			
0	10 (16.7%)	4 (21.1%)	14 (17.7%)
1	14 (28.0%)	6 (40.0%)	20 (30.8%)
2	21 (42.0%)	7 (46.7%)	28 (43.1%)
3	15 (30.0%)	2 (13.3%)	17 (26.2%)
**Premedication beta blocker**			
Missing data	1	0	1
Yes	53 (89.8%)	16 (84.2%)	69 (88.5%)
No	6 (10.2%)	3 (15.8%)	9 (11.5%)
**Premedication topiramate**			
Missing data	2	0	2
Yes	55 (94.8%)	17 (89.5%)	72 (93.5%)
No	3 (5.2%)	2 (10.5%)	5 (6.5%)
**Premedication flunarizine**			
Missing data	1	0	1
Yes	47 (79.7%)	15 (78.9%)	62 (79.5%)
No	12 (20.3%)	4 (21.1%)	16 (20.5%)
**Premedication amitriptyline**			
Yes	57 (95.0%)	19 (100%)	(96.2%)
No	3 (5.0%)	0 (0.0%)	3 (3.8%)
**Premedication valproate**			
Missing data	1	0	1
Yes	10 (16.9%)	3 (15.8%)	13 (16.7%)
No	49 (83.1%)	16 (84.2%)	65 (83.3%)
**Premedication OnabotulinumtoxinA**			
Missing data	3	11	14
Yes	52 (91.2%)	5 (62.5%)	57 (87.7%)
No	5 (8.8%)	3 (37.5%)	8 (12.3%)

*AMD* Monthly acute drug intake, *CM* Chronic migraine, *EM* Episodic migraine, *MHD* Monthly headache days, *MMD* Monthly migraine days, *MOH* Medication overuse headache, *SD* Standard deviation

**Fig. 2:**
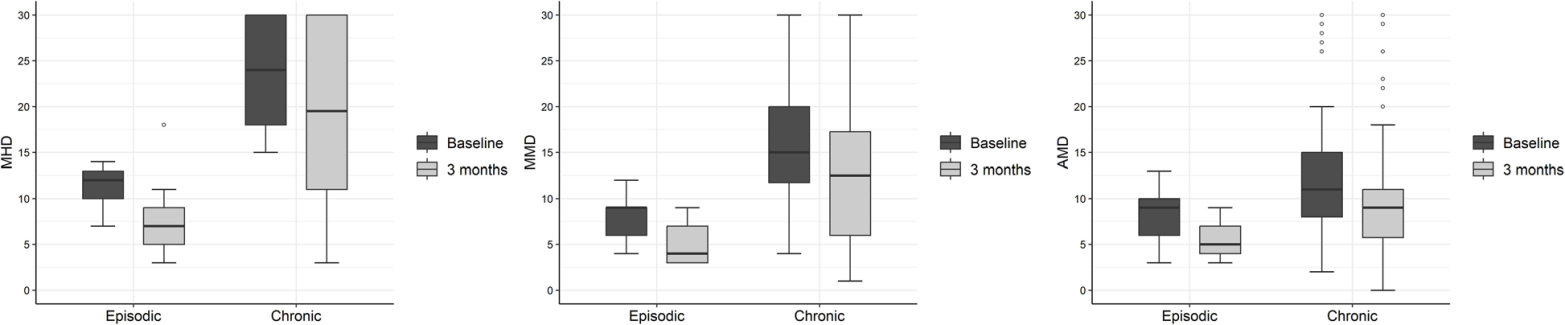
MHD, MMD and AMD at baseline and after three months of treatment. (AMD: monthly acute drug intake, MHD: monthly headache days, MMD: monthly migraine days)

For both EM and CM, a significant reduction in all parameters (MHD, MMD, and AMD) compared to baseline could be shown (Fig. 2, Table 2).

**Table 2:** Treatment response to eptinezumab after 3 months

			**Episodic (*N* = 19)**	**Chronic (*N* = 60)**	**Overall (*N* = 79)**
**MHD**	Baseline	Median (IQR)	12 (10.0, 13.0)	24 (18.0, 30.0)	20 (15.0, 29.0)
	Three months		7 (5.0, 9.0)	19.5 (11.0, 30.0)	14 (8.0, 27.5)
	Reduction		-4 (-6.5, -1.0)	-4 (-8.0, 0.0)	-4 (-8.0, 0.0)
	*p*-value		0.001	< 0.001	< 0.001
**MMD**	Baseline	Median (IQR)	9 (6.0, 9.0)	15 (11.8, 20.0)	12 (9.0, 17.0)
	Three months		4 (3.0, 7.0)	12.5 (6.0, 17.3)	8 (5.0, 15.0)
	Reduction		-3 (-5.5, -1.5)	-3 (-6.0, -1.0)	-3 (-6.0, -1.0)
	*p*-value		< 0.001	< 0.001	< 0.001
**AMD**	Baseline	Median (IQR)	9 (6.0, 10.0)	11 (8.0, 15.0)	10 (7.5, 13.0)
	Three months		5 (4.0, 7.0)	9 (5.8, 11.0)	8 (5.0, 10.0)
	Reduction		-2 (-5.0, -0.5)	-1 (-5.0, 0.0)	-2 (-5.0, 0.0)
	*p*-value		0.006	< 0.001	< 0.001

*AMD* Monthly acute drug intake, *CM* Chronic migraine, *EM* Episodic migraine, *IQR* Interquartile range, *MHD* Monthly headache days, *MMD* Monthly migraine days

Regarding EM patients, the 30% responder rate for MHD was 63.2% (12/19) and 57.9% (11/19) for MMD. The 50% responder rate for MHD was 36.8% (7/19) and for MMD was 42.1% (8/19). Regarding CM, the 30% responder rate for MHD was 35.0% (21/60) and 36.7% for MMD (22/60) (Table 3).

**Table 3:** 30% and 50% responder rates compared to baseline after 3 months

		Episodic (*N* = 19)	Chronic (*N* = 60)	Total (*N* = 79)
30% responder rate	MHD	12 (63.2%)	21 (35.0%)	33 (41.8%)
	MMD	11 (57.9%)	22 (36.7%)	33 (41.8%)
	AMD	8 (42.1%)	23 (38.3%)	31 (39.2%)
50% responder rate	MHD	7 (36.8%)	14 (23.3%)	21 (26.6%)
	MMD	8 (42.1%)	13 (21.7%)	21 (26.6%)
	AMD	6 (31.6%)	10 (16.7%)	16 (20.3%)

*AMD* Monthly acute drug intake, *MHD* Monthly headache days, *MMD* Monthly migraine days

**Fig. 3:**
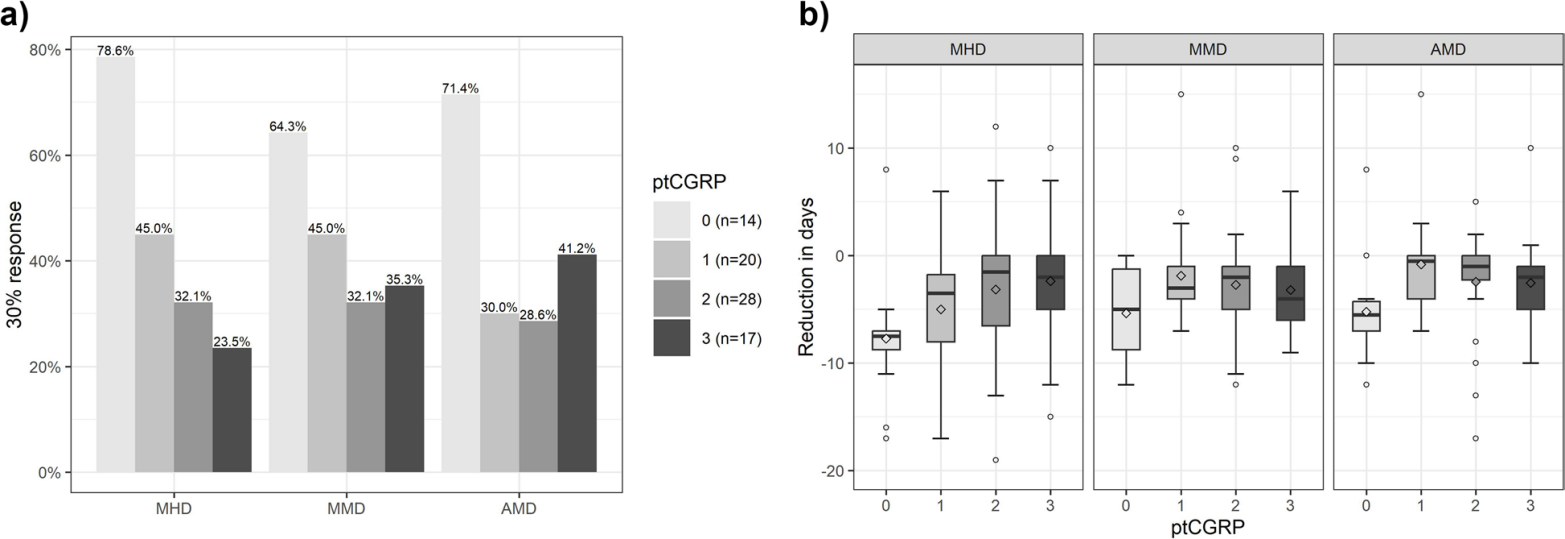
Response to eptinezumab after three months of treatment in dependency of CGRP (receptor) antibody pre-treatment. Responder rates (**a**) and reduction (**b**) depending on the number of prior CGRP mAb therapies. (AMD: monthly acute drug intake, CGRP: Calcitonin gene-related peptide, mAb: monoclonal antibody, MHD: monthly headache days, MMD: monthly migraine days, ptCGRP: pre-treatment with CGRP mAb (erenumab, galcanezumab or fremanezumab, ◊: mean)

In total, 14 patients were naïve to CGRP mAb, 20 patients had one, 28 patients had two and 17 had three ptCGRP. Responder rates of all patients who had at least one insufficient ptCGRP and naïve were analysed. The ptCGRP group had a lower 30% therapy response compared to naïve patients. The response depending on the number of ptCGRP is shown in Fig. 3. When comparing the 30% responder rates, there was a significant association between the number of ptCGRP and the 30% responder of MHD (Pearson's Chi-squared test: *p* = 0.010). However, the 30% responder rate of MMD was not significant (*p* = 0.225) and for AMD not significant after Bonferroni correction (*p* = 0.043).

Differences in response to eptinezumab in patients with only one ptCGRP (*n* = 20) were analysed descriptively according to their pre-treatment with different CGRP mab classes. Prior to eptinezumab treatment, ten patients received the CGRP receptor mAb (erenumab) and ten patients received a CGRP ligand mAb, either galcanezumab (*n* = 5) or fremanezunab (*n* = 5). Responder rates are shown in Table 4.

**Table 4:** 30% and 50% responder rates compared to baseline from patients with one CGRP mAb pre-treatment after 3 months

		Ligand (*N* = 10)	Receptor (*N* = 10)	Total (*N* = 20)
30% responder rate	MHD	6 (60.0%)	3 (30.0%)	9 (45.0%)
	MMD	5 (50.0%)	4 (40.0%)	9 (45.0%)
	AMD	3 (30.0%)	3 (30.0%)	6 (30.0%)
50% responder rate	MHD	2 (20.0%)	2 (20.0%)	4 (20.0%)
	MMD	3 (30.0%)	2 (20.0%)	5 (25.0%)
	AMD	0 (0.0%)	1 (10.0%)	1 (5.0%)

In the receptor group, all patients received erenumab in the past. In the ligand group, five patients received fremanezumab and galcanezumab, respectively

*AMD* Monthly acute drug intake, *MHD* Monthly headache days, *MMD* Monthly migraine days

Documentation of side effects was available for 77 patients. Eight patients (10.4%) reported mild side effects such as vertigo, constipation, rhinitis, myalgia, weight gain, hoarseness, and hypotension.

Satisfaction with therapy was documented for 70 patients, with 39 (55.7%) being very satisfied or satisfied, 15 (21.4%) being moderately satisfied, and 16 (22.9%) being unsatisfied or very unsatisfied with eptinezumab treatment.

## Discussion

Our data revealed a good effectiveness and tolerability of eptinezumab under real world conditions in therapy of patients, who are resistant to all conventional treatments. There was a significant reduction of MHD, MMD and AMD after three months of treatment. Side effects were rare and mild, 55.7% were satisfied or very satisfied with therapy. Nevertheless, the response was significantly reduced when patients had insufficient CGRP mAb therapies before eptinezumab. In patients with ptCGRP, a switch to eptinezumab led to a reduced response even after one ptCGRP. A significant association was found between the number of ptCGRP and the 30% MHD responder rate.

The effectiveness of eptinezumab was shown in the pivotal studies for approval. For EM patients, the reduction of MMD from baseline due to eptinezumab therapy (100mg) was 3.9 days during the first 12 weeks, 50% responder rate was 49.8% [1]. In the PROMISE-2 study, 50% MMD responder rate for CM was 57.6% during the first 12 weeks, MMD were reduced by 7.7 days [2]. In our cohort, 50% MMD responder rate was 42.1% for EM and 21.7% for CM and thus distinctly lower than in the approval studies. This might be due to our drug resistant cohort, even resistant to CGRP mAb therapy, indicating highly affected and hard to treat patients.

There is only weak evidence regarding real world effectiveness. A retrospective study was initiated in advisory boards for Lundbeck. Physicians who took part were recruited during these events. Eight physicians completed an online questionnaire for 31 patients about treatment with eptinezumab over 6 months. During 6 months of treatment, median MHD was reduced from 29 (interquartile range (IQR: 18.75 to 30)) to 15 days (IQR: 7.75 to 30), median MMD was reduced from 16 (IQR: 12 to 21) to 9.5 days (IQR: 3 to 12) after 6 months of treatment. AMD were reduced as well (12.5 (IQR: 9.5 to 17.9) before treatment, 7 (IQR: 1 to 12) after 6 months). No data on premedication were specified. Besides uncontrolled and/or untreated psychiatric conditions, another exclusion criterion was `a condition that, in the opinion of the clinician, would make them unsuitable for the clinical study´. Although the selection should not be affected by Lundbeck [11], a possible selection bias by the physicians themselves remains unclear.

A retrospective real-world study from the United Arab Emirates analysed 53 patients after three and six months of treatment. Altogether, 35 patients (66%) had a history of one failure and two patients (4%) had two failures of prophylactic treatment before. Patients mainly had CGRP mAB (galcanezumab, erenumab) as prior therapy, only four patients had other preventive treatments in the past. Eptinezumab let to a significant reduction after three and six months of treatment. The MMD response was remarkable for EM (75% and 50% MMD responder rate: 57.1% and 78.5% after 3 months; 57.1% and 82.1% after 6 months; respectively) and for CM (75% and 50% MMD responder rate: 32% and 60% after three months, 60% and 88% after six months; respectively). They found a significant difference in MMD after three months for patients who were naïve and for patients who were previously treated with erenumab or galcanezumab in (mean MMD after three months (SD): naïve: 2.13 (1.86); erenumab: 5.69 (5.96); galcanezumab: 7.47 (6.77), respectively; *p* < 0.001). After dosage increase from 100 to 300mg in 14 patients, MMD did not differ after six months (mean MMD after six months (SD): naïve: 3.25 (4.27); erenumab: 3.63 (4.86); galcanezumab: 3.82 (2.67)) [9]. The responder rates after 3 months were better than in our cohort, but after six months a remarkable better response was seen. This is contradictory to the data from the approval studies indicating an early response [12, 13]. Because we do not have data longer than three months, we do not know if our cohort would profit due to a dosage increase in the same manner.

CGRP mAb switch is a possible treatment option when patients are not responding to previous mAb. While erenumab targets the CGRP receptor, other available CGRP mAbs (including eptinezumab) bind the ligand CGRP itself. In real-world studies of erenumab non-responders, switching to a ligand mAb could improve treatment effectiveness [14]. However, the overall response rate was low (e.g. erenumab to galcanezumab or fremanezumab; MHD 30% response: 32%; MHD 50% response: 12% after three months [15]). In another real-world study, a similar effect was seen in the opposite direction in non-responders to galcanezumab/fremanezumab (switch to erenumab: MHD 30% response: 35%, 50% response: 5% [16]). In summary, the reduced response to the second CGRP mAb in previous studies is consistent with our data. Descriptively, the 30% response in all parameters was already reduced when eptinezumab was used as the second CGRP mAb (MHD: none: 78.6%, one ptCGRP: 45.0%; MMD: none: 64.3%, one ptCGRP: 45.0%; AMD: none: 71.4%, one ptCGRP: 30.0%). For MHD, a significant association between the number of ptCGRP and the 30% response was seen. Our data indicate a weaker response to eptinezumab when used as the second (third or fourth) CGRP mAb compared to patients who received eptinezumab as the first CGRP mAb (Fig. 3).

Although there is limited data on mAb switching between different classes, studies of mAb switching within the same class (ligand-to-ligand) are even rarer. Different molecular mechanisms have been assumed for each mAb class (e.g. internalisation of the CGRP receptor due to binding with erenumab [17] or different brain activity [18]), which could lead to a potential difference in response between the receptor and ligand mAbs. In a limited number of patients, a response was seen after switching from one ligand mAb to another, or even as a third mAb therapy, despite a previous class switch. However, these study groups were very small and it is not possible to generalise the results [19, 20]. In our study, 20 patients had one ptCGRP and received either erenumab (*n* = 10) or fremanezumab (*n* = 5)/galcanezumab (*n* = 5) prior to treatment with eptinezumab. Descriptively, patients who had previously received erenumab had even worse 30% responder rates and similar 50% responder rates compared to patients who had previously received a ligand mAb (Table 4). Our data do not suggest that patients who were previously non-responders to a ligand mAb are less likely to respond to eptinezumab, at least in this cohort. However, the groups compared are too small to make a valid statement.

Our analysis showed that eptinezumab was still effective in individual cases even after three failed ptCGRP, although a placebo effect of the infusion cannot be assessed due to the real-life setting. However, some patients did not respond at all. Our data support the hypothesis that there is a subpopulation of migraine patients who do not respond to CGRP mAbs in general and therefore do not respond to the second, third or fourth mAb. It is discussed that central sensitisation may play a key role (reviewed in [10]) and that it may not be driven, at least in part, by CGRP [21]. Therefore, CGRP mAbs may not be effective in this subgroup.

In addition to the aspect of effectiveness, the tolerability was good, even though eptinezumab is given as an infusion. Side effects were mild in our cohort and satisfaction with therapy was high. In PROMISE-1, 63.2% of EM patients reported, inter alia, side effects like upper respiratory tract infection, nasopharyngitis, dizziness, fatigue, cough and back pain [1]. In PROMISE-2, 43.5% of CM patients reported side effects like nasopharyngitis, upper respiratory tract infection, urinary tract infection, fatigue, sinusitis, migraine and nausea [2]. In our cohort, only 10.4% (8/77; NA = 2) reported side effects, which were partly different from those reported in the trials and included vertigo, constipation, rhinitis, myalgia, weight gain, hoarseness and hypotension. Taken together, these data also indicate that eptinezumab is a well-tolerated therapy under real-world condition despite the more elaborate way of application compared to the other CGRP mAbs.

Limitations of the study are the small sample size and missing data regarding long-term treatment effects. The short treatment period is also a relevant limitation, as 6 months of treatment are actually recommended to reliably evaluate the success of the therapy. On the other hand, early treatment response is considered to be a major advantage of the therapy with eptinezumab. In addition, there is an inequality of data acquisition in the respective centre (e.g. not standardised headache diaries, treatment documentation or physicians´ documentation). Moreover, no higher starting dosage (300mg) or increase to 300mg was performed. We also had no documentation about possible effects on acute migraine attacks. Thus, these possible effects of the treatment with eptinezumab cannot to be evaluated in this study. In addition, due to the nature of the application, placebo effects may be more pronounced than with subcutaneous injections [22]. Therefore, a strong placebo effect may interfere with the treatment effect and skew these real-world results.

## Conclusion

Our multicentre study supports the effectiveness of eptinezumab in preventive treatment of migraine under real-world conditions and provides evidence for good tolerability in patients resistant to conventional migraine prophylactic drugs. Nevertheless, treatment is distinctly less effective when patients were resistant to other CGRP mAb in the past. Despite previous failures in CGRP mAb treatment with up to three different antibodies, the therapy can still be effective on an individual basis. However, a placebo effect from the infusion itself can influence the treatment effect. Due to several limitations of the study (retrospective nature, small sample size, short treatment period with the lower dose), further studies with more patients are needed to evaluate the long-term treatment effect, dosage increase and effect on acute migraine attacks in order to establish the value of eptinezumab in clinical routine.

## Data Availability

The datasets used and/or analysed during the current study are available from the corresponding author on reasonable request.

## Abbreviations

AMD: Monthly acute drug intake
CGRP: Calcitonin gene-related peptide
CM: Chronic migraine
EM: Episodic migraine
IQR: Interquartile range
MHD: Monthly headache days
MMD: Monthly migraine days
mAb: Monoclonal antibody
MOH: Medication overuse headache
ptCGRP: Pre-treatment with CGRP (receptor) antibodies (erenumab, galcanezumab or fremanezumab)
